# Toward in vivo translation of super-resolution localization photoacoustic computed tomography using liquid-state dyed droplets

**DOI:** 10.1038/s41377-019-0171-9

**Published:** 2019-06-25

**Authors:** Wonseok Choi, Chulhong Kim

**Affiliations:** Departments of Creative IT Engineering, Electrical Engineering, Mechanical Engineering, Interdisciplinary Bioscience and Bioengineering, Pohang University of Science and Technology, Pohang, Gyeongbuk 37673 Republic of Korea

**Keywords:** Imaging and sensing, Photoacoustics

**Super-resolution localization photoacoustic computed tomography has been successfully performed in live animals using an injection of unprecedentedly absorptive liquid-state dyed droplets, which promises a disruptive modality for in vivo neuroimaging.**

Photoacoustic (PA; or optoacoustic) tomography is a popular imaging modality that forms an image from the received ultrasound signals generated from targets that absorb light. PA tomography achieves both optical absorption contrast and high ultrasonic spatial resolution even in deep tissues. Due to this property, PA tomography can be implemented to see objects on scales ranging from nanometers to millimeters at depths up to several centimeters^1^. Among the diverse implementations of PA tomography, PA computed tomography (PACT) is a dedicated modality for imaging deep tissues (>4 mm) in live animals, healthy humans, and patients. PACT typically uses array transducers in various geometries to receive PA signals and reconstruct them into images via acoustic inversion or beamforming techniques. Since PA wavefronts propagate in spherical shapes, circular or spherical arrays are regarded as the best signal reception geometries^2^. In addition, advanced electronics and processing units can perform fast image reconstruction that enables near real-time imaging. With these features, PACT systems are capable of imaging the whole body of small animals or providing diagnostic images for clinical applications, such as images of the breast.

In PA tomography, there is generally a tradeoff relationship between spatial resolution and imaging depth, where the resolution is typically on the order of 1/200 of the desired imaging depth for biological tissues^1^. For instance, PACT can usually be applied at depths up to several centimeters with a relatively poor resolution of a few hundred micrometers, which is determined by the size of the acoustic parameters. In other words, while PACT is capable of imaging at deeper depths, it comes at the cost of not being able to visualize detailed structures of the tissue, which are important for fully understanding their basic functional mechanisms.

New super-resolution imaging techniques are breaking through the physical resolution limitations mentioned above. To date, two mainstream super-resolution imaging techniques have been proposed for PACT: localization imaging and fluctuation imaging. Localization imaging is analogous to photoactivation localization microscopy or stochastic optical reconstruction microscopy, and fluctuation imaging is analogous to super-resolution optical fluctuation imaging. Localization super-resolution imaging has been demonstrated in vitro with 2D and 3D PA images by Vilov et al. and Deán-Ben et al., respectively, using sparsely distributed flowing microbeads (10–30 μm)^3,4^. They were successful in distinguishing tubes with diameters of approximately four times smaller than the diffraction limit. Chaigne et al. used the super-resolution fluctuation imaging technique, with PA signal fluctuation induced from varying speckle illumination and flowing absorbers, to calculate *n*-th order statistical values that enhanced the resolution by on the order of $\surd n$^5,6^. While both techniques have been demonstrated successfully in vitro, there have been no publications describing in vivo applications yet. Potential reasons for the lack of in vivo applications include low signal contrast in deep tissues, motion artifacts due to a limited frame rate, and the lack of appropriate isolated particles that can flow stably through the narrow vascular network.

To overcome these limitations for in vivo super-resolution imaging, Zhang et al. recently proposed dyed droplets as contrast agents for in vivo localization imaging^7^. Their key strategy is to use “liquid particles” that can smoothly flow through the blood vessels and generate prominent signal contrast, which is 300 times higher than the blood background. The dyed droplets were prepared by dissolving the IR-780 dye in oil and mixing the solution with water, which resulted in droplets with sizes ranging from 4 to 30 μm. For the in vivo demonstration in a mouse, the authors injected the dyed droplets through the left carotid artery of the mouse and used their ring-array-based PACT system (Fig. 1a) to image the live mouse brain. The dyed droplets generated high PA signal contrast sufficient for the signal to be localized, thus enabling the reconstruction of a super-resolution image (Fig. 1b). As a result, they were able to produce images of the neurovasculature up to a depth of 4 mm with a sixfold improvement in the spatial resolution compared to the original PACT images (Fig. 1c, d)^7^.

**Fig. 1:**
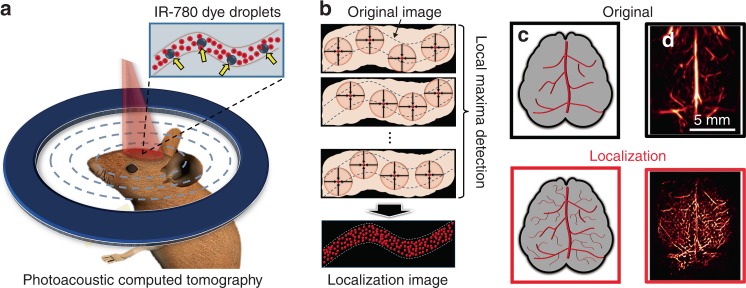
Principles of in vivo localization-based super-resolution photoacoustic computed tomography. **a** A photoacoustic computed tomography system acquires photoacoustic signals from blood vessels where IR-780 dye droplets are sparsely injected in vivo. **b** The droplets have 300-fold greater light absorption than the blood and thus appear as sparsely-distributed bright spots (orange circles) from which local maxima (red dots) can be detected in the acquired images. The localized maxima in a series of image frames are accumulated to create a localization image. **c**, **d** The localization image of the vasculature in the brain showed much more detailed structures than the original image, with a sixfold enhancement in the spatial resolution^7^

While Zhang et al.’s proposal mitigated the low signal contrast challenge and met the requirement of smoothly flowing particles, the biggest hurdle of a low frame rate is yet to be overcome. The frame rates for PACT are limited by low laser repetition rates, which are generally approximately 5–20 Hz, needed for a high-power laser to achieve better signal-to-noise ratios in the images. Zhang et al. used a PACT system with a frame rate of 20 Hz, and they noted that the speed was not sufficient to capture even slowly flowing droplets (1.3–7.5 mm/s)^7^. Thus, there exists a need to achieve a high frame rate for a sufficient signal-to-noise ratio in deep tissue imaging using a laser with sufficient power. As mentioned in Zhang et al.’s paper, there is still room to improve the in vivo localization technique by using a dye with a higher absorption coefficient and regulating the droplet size. Therefore, once the low frame rate issue is addressed, it is expected that we will see more in vivo demonstrations of super-resolution PACT in the near future.

## References

[1] Wang LV, Hu S (2012). Photoacoustic tomography: in vivo imaging from organelles to organs. Science.

[2] Choi W, Park E, Jeon S, Kim C (2018). Clinical photoacoustic imaging platforms. Biomed. Eng. Lett..

[3] Vilov S, Arnal B, Bossy E (2017). Overcoming the acoustic diffraction limit in photoacoustic imaging by the localization of flowing absorbers. Opt. Lett..

[4] Deán-Ben XL, Razansky D (2018). Localization optoacoustic tomography. Light. Sci. Appl..

[5] Chaigne T (2016). Super-resolution photoacoustic fluctuation imaging with multiple speckle illumination. Optica.

[6] Chaigne T, Arnal B, Vilov S, Bossy E, Katz O (2017). Super-resolution photoacoustic imaging via flow-induced absorption fluctuations. Optica.

[7] Zhang P, Li L, Lin L, Shi J, Wang LV (2019). In vivo super-resolution photoacoustic computed tomography by localization of single dyed droplets. Light. Sci. Appl.

